# Genomic Medicine and Individual Autonomy: Reflections on Knowledge Societies and Governmentality

**DOI:** 10.3390/ijerph23020234

**Published:** 2026-02-13

**Authors:** Richard H. Parrish

**Affiliations:** Alice L. Walton School of Medicine, Bentonville, AR 72712, USA; richard.parrish@alwmed.org; Tel.: +1-(706)-321-7218

**Keywords:** bioethical issues, genetic privacy, genomic medicine, government regulation, personal autonomy, social justice

## Abstract

**Highlights:**

**Public health relevance—How does this work relate to a public health issue?**

- Examines how genomic medicine and regulatory frameworks reshape disease classification, healthcare access, and surveillance with implications for autonomy and equity.
- Addresses genetic discrimination, compulsory treatment based on genetic risk, surveillance expansion, and pathologization of populations through risk identification in healthy individuals.

**Public health significance—Why is this work of significance to public health?**

- Highlights how genomic technologies influencing resource allocation, insurance, and treatment protocols could exacerbate disparities and undermine informed consent and autonomy.
- Identifies inadequate protections against genetic discrimination and coercive interventions, potentially creating genetic-based stratification threatening equitable healthcare access.

**Public health implications—What are the key implications or messages for practitioners, policy makers and/or researchers in public health?**

- Policymakers must develop governance balancing genomic benefits with protections for autonomy, privacy, and equity, including constitutional safeguards and data governance frameworks.
- Practitioners and researchers should recognize that genomic medicine introduces uncertainty for patients that requires participatory governance, dynamic consent, and reflexive regulation preserving autonomy while enabling beneficial applications.

**Abstract:**

This paper offers a comprehensive analysis of the multifaceted implications of genomic medicine’s evolving regulatory frameworks on individual autonomy. As genomic technologies increasingly permeate healthcare and society, they fundamentally reshape the boundaries of health and disease, profoundly impacting personal identity and self-understanding. The expansion of genomic surveillance and risk classification introduces new forms of scrutiny and vigilance, as individuals are redefined according to probabilistic genetic markers rather than traditional clinical symptoms. Regulatory developments facilitate compulsory interventions and challenge established notions of informed consent, as genetic risk factors in otherwise healthy individuals prompt preemptive medicalization and intervention. These changes heighten the risk of genetic discrimination and reinforce social stratifications, as access to care, insurance, and employment may become contingent upon genomic profiles. Furthermore, the commodification of genetic information raises significant concerns about privacy, ownership, and the potential misuse of personal data by commercial and governmental entities. The increasingly blurred lines between medical necessity and social control highlight constitutional and ethical dilemmas, particularly regarding the balance of public health priorities and the preservation of individual freedoms. Drawing on theoretical frameworks such as Stehr’s knowledge society and governmentality, the paper critically examines how regulatory responses both reflect and shape broader societal values, often introducing persistent uncertainty and vulnerability into the core of personal and collective identity. Ultimately, the analysis underscores the urgent need for innovative governance models that can effectively balance the promise of scientific and technological advances with the protection of personal autonomy, democratic knowledge control, and social justice in the genomic era. Lay statement: This paper explores how new rules and regulations around genetic medicine can impact people’s personal freedoms and sense of identity. It highlights concerns about privacy, discrimination, and the ways in which our understanding of health and disease is changing, calling for better protections and fairer policies as genetic technologies become more common.

## 1. Introduction

The complex intersection between genomic medicine, the application of genomic information and technologies to clinical care [1], and contemporary regulatory structures presents profound and far-reaching implications for individual autonomy that transcend traditional medical ethics considerations [2,3]. This comprehensive analysis examines how the evolving regulatory challenges associated with genomic medicine fundamentally reconfigure the relationship between individuals, their genetic information, and state authority, incorporating Nico Stehr’s influential theoretical perspectives on knowledge societies and the governance of knowledge within modern social structures [4,5,6,7,8,9,10,11].

Genomic technologies are fundamentally redefining health, disease, and human identity (See Appendix A for definitions of key terms) by transforming ordinary human conditions into medicalized treatable disorders. This alters how individuals conceive of their bodies, identities, and relationships to medical authority. This transformation is accompanied by an emerging genomic surveillance infrastructure that shifts medicine from a traditional symptom-based model to a preemptive intervention approach based on genetic predispositions, effectively creating a pre-symptomatic surveillance state.

This paradigm shift poses direct and concerning threats to individual autonomy in contemporary healthcare decision-making processes, particularly through the dramatic expansion of compulsory treatment justified by genetic risk (See Appendix A for definitions of key terms) by profiles rather than manifest symptoms in a phenomenon termed “pharmacogenomic paternalism.” Genomic medicine inevitably blurs the traditionally distinct boundaries between individual and collective interests, fundamentally challenging how regulatory systems conceptualize autonomous decision-making in healthcare contexts.

The ethical implications evoke historical parallels with eugenics, underscoring the enduring challenge of ensuring that advances in genetic science do not repeat the coercive and discriminatory practices of the past. Contemporary genomic regulatory frameworks risk creating a state-enforced dichotomous classification system dividing individuals into “incompetent self-medicators” versus “certified self-medicators,” where fundamental rights become contingent on proper management of one’s genetic identity rather than being recognized as inherent.

These developments accelerate the commodification of genetic information and transform it from an intrinsic aspect of personal identity into intellectual property. Current legal structures appear fundamentally inadequate to address how genomic medicine transforms the relationship between individuals and the state regarding bodily autonomy and medical decision-making.

Preserving meaningful autonomy in this genomic era may require explicit constitutional protection of self-determination in medical contexts that specifically address genomic information, as ordinary legislation or executive orders remain too susceptible to political pressures and shifting policy priorities to provide enduring safeguards against governmental overreach. (See Supplementary Material, File S1: Generative AI Use Statement.)

## 2. The Paradigmatic Redefinition of Health, Disease, and Human Identity

The genomic era introduces unprecedented classifications that transform previously normal human variations into medical conditions and fundamentally alter conceptualizations of health, disease, and identity [12,13]. As genomic technologies advance, the boundary between biological diversity and treatable medical conditions becomes increasingly contested. Conrad [14] documents how genomic technologies accelerate medicalization and alter how individuals conceptualize and experience their own bodies and identities.

Juengst [15] argues that genomic medicine threatens to pathologize the entire population by identifying genetic risk factors in asymptomatic, healthy individuals. This classification power reshapes self-understanding, as genetic variations previously considered normal become redefined as medical conditions requiring intervention [16]. Stehr’s [4] concept of “knowledge societies” illuminates this transformation. Contemporary societies increasingly structure themselves around specialized knowledge production rather than material goods. Genomic medicine exemplifies this shift, as genetic knowledge becomes primary for defining health and illness parameters [17,18].

These transformations create new forms of social and individual vulnerability [19]. Individually, constantly redefined boundaries of “normal” destabilize identity formation.

Genetic traits once benign may suddenly become disease risk factors, causing confusion and anxiety [20]. The APOE ε4 allele exemplifies this: Once considered benign, it is now a significant Alzheimer’s disease risk factor [21]. Carriers face increased medical scrutiny and anxiety despite being asymptomatic, transforming neutral identity aspects into surveillance sources [20]. Alzheimer’s disease prevalence is projected to increase substantially as populations age and genomic medicine continues to refine risk stratification methods. Genomic research has identified numerous risk factors, refining prevalence estimates and understanding population vulnerability [21]. As Conrad [14] and Stehr [4] discuss, genomic advancements increase projection accuracy and influence disease classification and management, shaping future diagnosis, prevention, and intervention approaches.

Societally, uncertainty emerges as understandings of health, disease, and acceptable variation rapidly change. Regulatory frameworks, medical guidelines, and insurance policies must adapt quickly, creating inconsistencies or gaps [22]. Constant renegotiation of treatable or risky conditions may fragment society as communities interpret shifting norms divergently [12,13].

Genomic information’s probabilistic nature compounds this uncertainty [23]. Individuals grapple with ambiguous risk assessments that do not guarantee disease development but influence life decisions, family planning, employment, and insurance eligibility [20]. This probabilistic nature erodes trust in medical authority as individuals navigate complex, uncertain landscapes with limited information [16].

Stehr’s [4] insight highlights how technological innovation and regulatory adaptation in genomic medicine reconfigure societal norms and introduce persistent uncertainty into personal identity and social organization. This uncertainty, while sometimes catalyzing progress, can engender vulnerability, instability, and alienation as individuals and societies adjust to new definitions of normality and risk [16,19]. The dynamic interplay between genomic knowledge production and its societal application necessitates careful consideration of equity, access, and the potential for both benefit and harm across diverse populations [22].

## 3. The Systematic Emergence of Genomic Surveillance Infrastructure

The anticipated future trajectory of medicine increasingly includes sophisticated treatment regimens consisting of precisely tailored pharmaceuticals administered by specialized paraprofessionals based specifically on an individual’s comprehensive genomic profile [24,25]. This development represents a profound paradigmatic shift from the traditional medical model of treating manifest symptoms to a preemptive intervention approach based on genetic predispositions that may never manifest clinically. This fundamental shift creates what Prainsack and Reardon [26] have characterized as “genetic surveillance,” where regulatory frameworks enable continuous monitoring of genetic risk factors across populations.

Recent large-scale genomic initiatives demonstrate both the clinical utility and surveillance implications of population-wide genomic sequencing. The UK’s 100,000 Genomes Project pilot study achieved genetic diagnoses in 25% of participants with rare diseases, with 25% of these diagnoses having immediate clinical actionability [27]. Similarly, comprehensive metabolomic profiling of 250,341 UK Biobank participants identified 54 aging-related biomarkers predictive of mortality and disease risk, demonstrating superior short-term mortality prediction compared to chronological age alone [28]. These advances in genomic and metabolomic surveillance capabilities raise fundamental questions about medical privacy, confidentiality, and individual autonomy in healthcare decision-making processes [2,29].

Moreover, while biobanking enables genome-targeted therapies, for example, on cancer and rare diseases, it also raises concerns regarding privacy protection, disclosure of sensitive health information, and the transfer of data to external institutions such as governmental, insurance, and commercial entities, which may give rise to fears of discrimination against donors. Domaradzki and colleagues [30] identify these risks in a survey of 548 Polish hospitalized patients where concerns about data security and confidentiality were expressed by 41.8% of respondents, and 47.1% reported concerns about unethical use of their data. Most frequently, respondents indicated fear of the use of biological samples in commercial research for profit-making purposes (51.1%). Additionally, one-fifth of patients were concerned about the risk of stigmatization and discrimination. However, very few patients indicated distrust of science, scientists, and research institutions (7.3%), implying that the fear of data misuse is linked directly to corporate interests.

Consumer genomic testing (CGT), encompassing both direct-to-consumer (DTC) and consumer-initiated testing, exemplifies the expansion of genetic surveillance beyond traditional clinical settings. A survey of 139 genetics healthcare providers revealed that 94% encountered challenges counseling patients after CGT, including adverse psychosocial events (76%), incorrect variant interpretation (68%), and unconfirmed results (69%) [31]. These findings underscore concerns about clinical utility and potential harms when genetic surveillance occurs outside established regulatory frameworks. As Rothstein [32] argues, “Genomic medicine creates unprecedented opportunities for surveillance of individuals based on their genetic makeup,” fundamentally challenging traditional conceptions of medical privacy, confidentiality, and autonomy.

Stehr’s groundbreaking work on “knowledge politics” [10] is particularly relevant to understanding this surveillance dynamic. Stehr persuasively argues that as knowledge becomes increasingly central to governance structures, new forms of surveillance inevitably emerge that monitor not just observable behaviors but potential future behaviors and health outcomes. Genomic surveillance represents perhaps the ultimate manifestation of this knowledge-based monitoring approach, as it focuses on genetic potentialities rather than clinically manifest conditions, creating what might be termed a “pre-symptomatic surveillance state” with profound implications for individual privacy and autonomy [33,34].

## 4. The Expansion of Compulsory Treatment Based on Genetic Risk Profiles

The regulatory apparatus of the state could eventually declare conditions such as obesity, addiction, or cognitive variations as dangerous public health hazards requiring systematically monitored dietary regimens, pharmaceutical interventions, and ongoing therapies specifically designed to assuage genetic profiles deemed defective or suboptimal. This regulatory approach would extend beyond current practices of involuntary treatment beyond the mental health domain into other medical specialties based primarily on genomic risk factors rather than manifest symptoms. The potential for genomic medicine to dramatically expand the scope of compulsory treatment represents one of the most direct and concerning threats to individual autonomy in contemporary healthcare decision-making processes [2,3,12,35].

Recent research demonstrates that polygenic scores (PGS) for body mass index (BMI) can predict obesity risk across diverse populations and throughout the life course, with explained variance ranging from 2.2% in rural Ugandans to 17.6% in UK Biobank participants of European ancestry [36]. While these scores show promise for early risk identification, their implementation raises concerns about potential misuse in justifying compulsory interventions. Individuals with higher PGS showed accelerated BMI gain from age 2.5 years and greater adult weight gain, suggesting genetic predisposition manifests early and persists throughout life.

However, other recent evidence challenges the notion that genetic predisposition is immutable. Kim et al. [37] demonstrated that adherence to healthy lifestyles can offset genetic risk for obesity, with lifestyle factors nearly doubling explained variance for BMI from age 5 onward. Individuals with high genetic risk who maintained healthy lifestyles prevented obesity-related morbidities, supporting the view that genetic risk is modifiable through environmental intervention rather than requiring compulsory medical treatment. Among participants in intensive lifestyle intervention trials, those with higher genetic predisposition lost modestly more weight initially but were more likely to regain it, suggesting genetic susceptibility influences both weight loss and maintenance responses.

Furthermore, the framing of neurodevelopmental variations as “disorders” requiring intervention is increasingly challenged. Swanepoel [38] argues that ADHD and autism represent normal biological variations rather than pathological conditions, as evolutionary science suggests these traits conferred adaptive advantages in ancestral environments. This perspective aligns with the neurodiversity movement, which views variations in brain function as normal population diversity rather than disorders requiring correction. The author emphasizes that “a person can only be said to be mentally ill or disordered if that person experiences suffering or distress to a considerable extent,” which cannot be said for many neurodiverse individuals who lead successful lives.

Expert knowledge increasingly serves as justification for compulsory interventions across various domains of social life. Genomic medicine amplifies this dynamic by providing seemingly objective genetic markers that experts can utilize to justify intervention, potentially overriding individual preferences [9]. As the authority of science transforms into the authority to govern, new forms of knowledge-based compulsion operate under the guise of scientific objectivity while potentially serving social control functions.

## 5. The Fundamental Transformation of Informed Consent in Genomic Contexts

Traditional informed consent models face increasing challenges in genomic medicine contexts [39,40]. Genomic information fundamentally challenges traditional consent models because its implications extend far beyond the individual to biological family members, future generations, and potentially entire population groups sharing genetic characteristics. This paradigmatic shift from individual to communal concepts of autonomy represents a central ethical challenge in genomic medicine regulation. Genomic medicine inevitably blurs the traditionally distinct boundaries between individual and collective interests, which fundamentally challenges how regulatory systems conceptualize autonomous decision-making in healthcare contexts [41].

How this transformation will unfold over time and affect patients can be estimated from studying future healthcare professionals. Knowledge and attitudes about informed consent regarding biobanking from a survey of 865 Polish medical, nursing, and pharmacy students [42] revealed that while 70.9% would donate their biological material, there was a variety of attitudes toward consent. More than half the students (51.9%) opted for a study-specific, 16.6% for blanket, and about 10% for broad consent. A majority (89.9%) believed that donors’ biological material should be reversibly coded, not anonymized. Almost all students were interested in knowing the type and purpose of the study they would be donating and who would have access to the research results. Significant differences were found in students’ opinions on the control over data sharing. These data mirror patients’ concerns regarding unethical and unauthorized commercial use despite giving specific consent.

Stehr’s sophisticated analysis of the “governance of knowledge” provides important contextual understanding for this challenge. In “Who Controls Knowledge?” [6], Stehr comprehensively examines how the production and distribution of specialized knowledge create new governance challenges that traditional regulatory models are fundamentally ill-equipped to address. Recent WHO guidance introduces a “granularity maximization” principle requiring informed consent to be as detailed as possible regarding potential data uses, benefits, risks, and infrastructure. However, this approach conflicts with established data protection frameworks that balance individual privacy rights with societal benefits [40]. Excessive detail risks undermining participant autonomy through information overload, as research consistently demonstrates that overwhelming participants with excessive detail diminishes their capacity to make informed choices [43]. A participant-centered materiality standard focusing on information that reasonable research participants would find material to their decision may represent a more effective alternative [40]. Genomic information exemplifies what Stehr characterizes as “actionable knowledge” with implications that extend far beyond conventional individual decision-making frameworks that requires new conceptual approaches to informed consent that acknowledge its inherently relational nature [3,44].

## 6. The Multifaceted Emergence of Genetic Discrimination (See Appendix A for Definitions of Key Terms) Despite Regulatory Protections

While legislative frameworks such as the Genetic Information Nondiscrimination Act (GINA) [45] ostensibly aim to prevent discrimination based on genetic information, genomic medicine creates subtle forms of discrimination that contemporary regulatory frameworks struggle to adequately address [46,47]. The non-utilization of available genome-based therapies might be viewed as irresponsible, particularly when individual decisions potentially affect future generations or public health outcomes. Rothstein [48] cautions that “genetic exceptionalism” within regulatory frameworks may paradoxically increase stigmatization by treating genetic information as fundamentally different from other categories of health information, creating what has been termed “genetic determinism”, where genetic factors receive disproportionate weight in determining individual rights, responsibilities, and access to resources.

The expansion of compulsory treatment and regulatory surveillance based on genomic risk profiles bears striking similarities to the eugenics movement that gained prominence in the early twentieth century [49]. Both frameworks rely on scientific authority to justify state intervention often under the guise of public health. In the eugenics era, policies such as forced sterilization were implemented to “improve” the genetic quality that frequently resulted in the erosion of personal autonomy and the marginalization of vulnerable groups. Contemporary genomic medicine, while technologically advanced, echoes these dynamics by enabling forms of surveillance and intervention that may override personal choice in the name of genetic optimization [50]. The parallels underscore the enduring ethical challenge of ensuring that advances in genetic science do not repeat the coercive and discriminatory practices historically associated with eugenics.

From a medical perspective, the concept of normality, referred to earlier in this commentary, from a eugenics context historically centers on defining biological standards that distinguished “fit” from “unfit” individuals. Modern medical ethics has largely rejected eugenic frameworks, yet tensions persist around genetic screening and enhancement technologies. Contemporary bioethics scholars argue that medical definitions of normality remain contested, particularly as prenatal testing and gene editing technologies like CRISPR raise questions about which traits constitute disease versus difference [51]. The medicalization of disability through a eugenic lens has been critiqued for pathologizing natural human variation rather than recognizing diverse embodiments as normal [52]. Current debates surrounding PGR scores and embryo selection reveal how genetic technologies risk reinscribing eugenic logic under the guise of preventive medicine, where statistical deviation from population norms becomes conflated with medical abnormality [53].

Socially, the eugenics movement constructed normality through racialized, ableist, and classist hierarchies that justified reproductive control over marginalized populations. Contemporary scholars emphasize how eugenic ideologies persist in implicit biases within healthcare systems and reproductive counseling [54]. Cultural constructions of normality reflect dominant group values rather than objective standards, with disability studies scholars arguing that social arrangements, not bodily differences, create disadvantage [55]. The concept of “normal” serves as a regulatory mechanism that polices boundaries of acceptable human variation and reveals how eugenics functioned less as science than as ideology reinforcing existing power structures [56].

Despite GINA’s protections in employment and health insurance, significant gaps remain [57]. The legislation does not cover life insurance, disability insurance, or long-term care insurance, and protects only asymptomatic individuals. Moreover, legal protections vary significantly by jurisdiction, with some countries adopting comprehensive human rights-based approaches while others rely on industry moratoria or existing privacy laws [46]. The evolving nature of genetic technologies, including whole genome sequencing and epigenetic testing, challenges existing legal frameworks that may not adequately cover these newer forms of genetic information.

Stehr’s [8] concept of “moral markets” offers insights into this discriminatory dynamic. In “Moral Markets,” Stehr examines how knowledge societies increasingly moralize economic transactions and resource allocation decisions. Genomic medicine creates what might be termed “moral genomics,” where genetic decisions become subject to moral evaluation and potential discrimination based on compliance with emerging genomic norms. As Stehr notes, “knowledge societies moralize markets,” and genomic medicine similarly moralizes genetic variation, creating new forms of discrimination that operate under the guise of objective science [8].

## 7. The Problematic Dichotomization of Genomic Autonomy

A particularly concerning potential development within genomic regulatory frameworks is the emergence of a state-enforced dichotomous classification system dividing individuals into “incompetent self-medicators” versus “certified self-medicators.” Genomic medicine accelerates this bifurcation process by providing ostensibly objective genetic markers that can be utilized to justify differential treatment of individuals based on their presumed capacity to understand and appropriately respond to genetic information [58]. The “certified self-medicator” in genomic contexts would be someone who has received formally approved training, registered with appropriate regulatory authorities to possess medication, and maintains membership in recognized patient advocacy groups [59]. This regulatory approach creates what Rose [34] calls “biological citizenship,” where fundamental rights become contingent on proper management of one’s biological (and specifically genetic) identity rather than being recognized as inherent [60].

The integration of pharmacogenomics into clinical practice exemplifies these emerging divisions. Haidar et al. [61] demonstrated that preemptive pharmacogenomic testing requires substantial infrastructure, creating potential barriers for patients who lack access to institutions with such resources or who cannot navigate complex genomic information independently. Furthermore, Boardman et al. [62] found significant differences between the general population and families with direct experience of genetic conditions regarding attitudes toward carrier screening, highlighting how genomic decision-making increasingly occurs in contexts where individuals lack the experiential framework to fully understand the implications of genetic information.

The global adoption of genomic medicine further exacerbates these divisions. Chediak et al. [63] emphasize that socio-cultural factors, including stigma, religious beliefs, and educational disparities, create significant barriers to genomic medicine access in low- and middle-income countries. The lack of diverse genetic reference databases means that genomic testing may be less accurate for underrepresented populations, creating a two-tiered system where genomic “competence” is partially determined by one’s ethnic and geographic background.

The emerging landscape of personalized genomic medicine thus risks creating a system where fundamental rights become contingent on proper management of one’s biological identity [64]. As genomic data becomes increasingly central to healthcare decision-making, those unable to access, interpret, or act upon such information face systematic disadvantages, potentially entrenching new forms of inequality based on genomic literacy and access. Stehr’s [7] analysis of knowledge inequality is particularly relevant to understanding this trend. Genomic medicine intensifies this stratification process by creating “knowledge classes,” that is, those who can effectively navigate complex genomic information versus those who cannot. This stratification directly impacts individual autonomy, as those deemed genomically “incompetent” face increasing restrictions on their healthcare decision-making capacity [2,3].

## 8. The Accelerating Commodification of Genetic Information

Genomic medicine creates unprecedented forms of intellectual property requiring comprehensive regulatory frameworks. Effective policy approaches should prioritize clearly defined patents and property rights with protection of participant autonomy and data sharing imperatives. Recent scholarship highlights “the commodification of the body,” where genetic information becomes conceptualized as property rather than an intrinsic aspect of personal identity [65]. This commodification fundamentally challenges traditional notions of bodily autonomy and self-ownership that have historically underpinned medical ethics [66].

The rise of DTC genetic testing services has further complicated the commodification landscape, as commercial entities increasingly market genetic information as a consumer product while simultaneously claiming proprietary rights over aggregated genetic databases. This dual positioning, that is, treating genetic information as both personal property that individuals can purchase insights about and corporate assets that companies can monetize, creates fundamental contradictions in how autonomy and ownership are conceptualized in the genomic era. The CRISPR patent disputes exemplify these tensions, demonstrating how intellectual property conflicts among academic institutions raise concerns about access, innovation, and ethical governance [31].

Genetics healthcare providers increasingly encounter challenges when counseling patients who have received consumer genomic testing results. A 2024 survey found that 94% of providers faced at least one challenge, including adverse psychosocial events (76%), incorrect variant interpretation (68%), and unconfirmed results (69%). Providers reporting higher challenge frequencies were more likely to indicate that harms of consumer genomic testing outweigh benefits, highlighting the practical consequences of inadequate regulatory oversight [31].

Patent protection for genetic technologies presents additional ethical dilemmas. Patents can serve as tools for private governance, potentially restricting harmful applications or ensuring equitable access. However, they may also impede scientific collaboration and concentrate control in the hands of commercial surrogates rather than research institutions. The challenge of balancing proprietary interests with the public good remains central to genomic medicine’s future trajectory [67].

Contemporary informed consent frameworks struggle to address these complexities. The 2024 WHO guidance advocates for “granularity maximisation” in consent processes, yet this approach risks information overload and may conflict with established data protection principles emphasizing sufficiency rather than exhaustiveness [40]. A participant-centered materiality standard by focusing on information a reasonable research participant would find material to their decision that offers a more balanced approach that respects autonomy without overwhelming participants [40].

Stehr’s groundbreaking work on the “knowledge economy” provides essential context for understanding this commodification process [5]. In “Knowledge Societies,” Stehr systematically examines how knowledge becomes a primary economic resource in advanced societies, creating new forms of value and ownership. Genomic information exemplifies what Stehr characterizes as “knowledge as property,” creating unprecedented tensions between individual ownership of genetic information and corporate ownership of therapeutics derived from that information. As Stehr notes, “the appropriation of knowledge becomes a central conflict in knowledge societies,” [5] and genomic information represents perhaps the most personal form of knowledge that can be appropriated [18].

## 9. The Fundamental Challenge to Constitutional Protections

Existing constitutional frameworks may inadequately protect individuals from increasingly intrusive forms of forced therapeutic interventionism in genomic contexts. Current legal structures fundamentally fail to address how genomic medicine transforms the relationship between individuals and the state regarding bodily autonomy and medical decision-making [68]. Jones et al. [69] argue that reproductive autonomy in the genomic era requires a relational framework that balances individual choice with structural influences, suggesting that constitutional-level protections are necessary to safeguard self-directed medical decision-making. A United States federal constitutional amendment specifically guaranteeing the right of genomic privacy would establish fundamental rights that ordinary legislation or executive orders fundamentally cannot achieve due to its susceptibility to political pressures and shifting policy priorities.

The intersection of genomic information with law enforcement and criminal justice systems presents additional constitutional concerns. Forensic genetic databases, familial searching techniques, and the expansion of DNA collection from arrestees raise profound United States Fourth Amendment questions about unreasonable searches and seizures [70]. Hazel and Slobogin [70] empirically demonstrate that law enforcement access to genetic databases from healthcare providers and DTC testing companies should require prior judicial authorization. Ram [71] identifies critical gaps in statutory protections around law enforcement use of genetic biobanks, recommending tightened consent requirements to protect genetic privacy while promoting public safety. Jung et al. [72] proposed cryptographic frameworks for encrypting DNA fingerprints to safeguard privacy in forensic contexts.

Stehr’s [10] analysis of “knowledge politics” offers particularly valuable insight into this constitutional challenge. In “Knowledge Politics,” Stehr comprehensively examines how knowledge-based governance creates unprecedented challenges for democratic institutions designed in pre-genomic eras. Genomic medicine exemplifies what Stehr characterizes as the “constitutionalizing of knowledge,” where fundamental questions about knowledge production and application require constitutional-level responses rather than merely regulatory adjustments to existing frameworks. The challenge of balancing privacy risk with data utility in personalized medicine contexts demands governance frameworks that protect human rights while enabling responsible innovation [68,73].

## 10. The Problematic Emergence of Pharmacogenomic Paternalism

Genomic medicine enables what scholars term “pharmacogenomic paternalism,” where regulatory frameworks increasingly justify intrusive interventions based on genetic risk profiles rather than manifest symptoms. This represents a significant evolution of traditional medical paternalism from “doctor knows best” to “your genome knows best,” with regulatory systems enforcing compliance with genomically determined treatment protocols that may override individual preferences and values [74]. This fundamental shift challenges core bioethical principle of respect for autonomy, as genetic determinism provides seemingly objective scientific justification for overriding individual preferences regarding healthcare decisions. The substantial risk is creating a regulatory system where personalized medicine paradoxically diminishes personal freedom through increasingly granular control mechanisms justified by genomic knowledge [3].

The implementation of pharmacogenomic testing in clinical settings has begun to reveal tensions between evidence-based protocols and patient autonomy. Recent research demonstrates that approximately one-quarter of cancer patients carry disease-relevant pharmacogenomic variants, with those possessing toxicity-associated genotypes experiencing significantly higher rates of drug-related adverse effects than wild-type counterparts [75]. When genetic testing indicates that a patient is likely to experience adverse reactions to a standard medication or may not metabolize a drug effectively, healthcare providers face ethical dilemmas about whether to mandate alternative treatments, even when patients may prefer to try standard therapies. These situations highlight how genomic knowledge can be used to constrain rather than expand patient choice, creating what might be termed “genetic essentialism” in clinical decision-making. Moreover, the increasing integration of electronic health records with genomic databases creates infrastructure for systematic monitoring of compliance with genomically informed treatment recommendations. This technological capability raises concerns about the potential for “algorithmic paternalism,” where automated systems flag non-compliance with genomic protocols, potentially triggering interventions that further erode patient autonomy.

Stehr’s concept of “knowledge-based authority” provides essential context for understanding this paternalistic dynamic. In “Knowledge and Power” [11], Stehr systematically examines how specialized knowledge creates new forms of authority in advanced societies that operate alongside traditional authority structures. Genomic medicine exemplifies what Stehr characterizes as “epistemic authority,” where specialized knowledge of genetic risk factors becomes sufficient justification for paternalistic intervention in individual healthcare decisions. As Stehr perceptively notes, “knowledge societies generate new forms of authority based on claims to superior knowledge,” [11] and genomic medicine represents perhaps the ultimate claim to superior knowledge about an individual’s health trajectory.

## 11. Conclusions: Genomic Medicine and Individual Autonomy: Toward Comprehensive Ethical Frameworks

Contemporary genomic medicine presents unprecedented challenges to individual autonomy, demanding fundamental reconsideration of regulatory principles developed in pre-genomic contexts [76]. As Stehr [4] observes, “The fragility of modern societies is rooted in their dependence on knowledge,” an insight particularly relevant to genomic medicine, which creates powerful new forms of knowledge that can either substantially enhance or significantly diminish human autonomy depending on how regulatory frameworks are constructed. The COVID-19 pandemic highlighted ethical tensions between digital surveillance technologies and privacy protections, revealing how rapidly evolving data collection methods can outpace traditional consent frameworks [76].

Digital epidemiology and genetic research increasingly rely on data sources generated outside traditional healthcare systems, including social media, mobile devices, and electronic health records [76]. These technologies enable faster disease detection and broader population representation but introduce validity concerns, algorithmic biases, and risks of stigmatization when non-health data are presented as risk factors [76]. Privacy infringements occur when personal data from cell phones, geolocation apps, and social media are shared with governments for COVID-19 alerts, mobility tracking, and quarantine enforcement without informed consent [76].

Genetic epidemiology research exemplifies these challenges through large-scale prospective cohort studies utilizing whole-genome sequencing, electronic health records, and real-time behavioral monitoring [76]. While offering potential health benefits through clinically actionable results and risk factor modification, participation entails substantial risks including unintended data access through hacking or triangulation, permitted but unwanted secondary uses, and psychological harm from uncertain genetic results [76]. Hereditary cancer patients face cascading privacy risks throughout kinship networks, as relatives may share genomic information enabling reidentification despite anonymization efforts [44].

Reidentification can occur through triangulation of publicly available data with research datasets, matching three-dimensional face maps, inference by kinship relationships, or variant rarity [44]. The human genome constitutes the ultimate personal identifier, making anonymity difficult to ensure despite promising medical applications [45]. Ethical frameworks must address these interconnected challenges through what has been characterized as “reciprocity governance,” recognizing both individual and collective interests in genomic information without subordinating individual autonomy to population health objectives [76].

Stehr’s [10] concept of “knowledge democracy” offers a valuable framework for addressing these complex challenges. In “Knowledge Politics,” Stehr argues that knowledge societies require new democratic institutions specifically designed to govern knowledge production and application while preserving individual autonomy within increasingly knowledge-based social structures. For genomic medicine, this suggests creating governance frameworks that preserve individual autonomy while enabling beneficial applications of genomic knowledge through participatory mechanisms rather than expert-dominated regulatory structures, what Stehr characterizes as “democratizing knowledge” [10].

Emerging governance models offer promising directions for balancing competing interests. Dynamic consent allows individuals to modify preferences over time as circumstances and research landscapes evolve, addressing temporal and relational complexities of genomic information [76]. Participatory governance involving patients, community representatives, and diverse stakeholders in research oversight ensures regulatory frameworks reflect broader values rather than privileging institutional interests [76]. Robust data governance frameworks must protect privacy while enabling beneficial research, addressing initial collection, secondary uses, data sharing across jurisdictions, and individual rights to access or withdraw genetic information [76].

International harmonization presents opportunities for preventing regulatory arbitrage while accommodating legitimate cultural differences regarding genetic information, family relationships, and balancing individual versus collective interests [76]. The development of flexible frameworks that establish core principles while allowing contextual adaptation may offer the most promising path forward, requiring what Stehr [10] terms “reflexive governance,” that is, governance structures that continuously reflect on their own knowledge claims and their implications for human freedom and self-determination in healthcare contexts.

Only through thoughtfully designed regulatory innovation through durable constitutional processes can society ensure genomic medicine enhances rather than diminishes human autonomy. This requires sustained attention to power dynamics embedded in genomic knowledge production, critical examination of how frameworks may reproduce social inequalities, and genuine commitment to centering individual autonomy and dignity within governance structures [76]. As genomic technologies evolve rapidly around the world, regulatory frameworks must remain adaptive while maintaining core commitments to human rights, social justice, and meaningful autonomy in an increasingly genomically defined world.

## Data Availability

No new data were created or analyzed in this study.

## Supplementary Materials

The following supporting information can be downloaded at: https://www.mdpi.com/article/10.3390/ijerph23020234/s1, File S1: Generative AI Use Statement.

## Appendix A. Definitions of Key Terms

- Health is a dynamic state of complete physical, mental, and social well-being, not merely the absence of disease or infirmity, encompassing the capacity to adapt and self-manage in the face of social, physical, and emotional challenges [77].
- Disease is a pathological condition characterized by identifiable structural or functional abnormalities in the body that disrupts normal physiological processes and manifests through specific signs, symptoms, or diagnostic markers [78].
- Human identity is the multifaceted construct encompassing an individual’s sense of self, formed through the integration of biological, psychological, social, and cultural dimensions, including continuity of personal narrative, embodied experience, relational connections, and membership in social categories [79].
- Genetic risk is the probability that an individual will develop a particular disease or condition based on their genetic makeup, determined through analysis of specific gene variants, mutations, or polygenic scores that confer increased susceptibility compared to the general population [80].
- Genetic discrimination is the differential treatment of individuals or their relatives based on actual or perceived genetic characteristics, occurring when genetic information is used to deny opportunities, services, or benefits in contexts such as employment, insurance, education, or healthcare [81].
