# Supplementary material for: Genomic Medicine and Individual Autonomy: Reflections on Knowledge Societies and Governmentality
Source: Int J Environ Res Public Health. 2026 Feb 13;23(2):234. doi: 10.3390/ijerph23020234 (PMC12941236; doi:10.3390/ijerph23020234)
Supplement: Supplementary file 1 [file ijerph-23-00234-s001.zip › ijerph-4130583-supplementary.pdf]

Generative AI Use Statement

Genomic Medicine and Individual Autonomy: Reflections on Knowledge Societies and Governmentality

During the preparation of this work, the author used Claude 4.5 Sonnet (Anthropic) to assist with literature review and reference formatting. Specifically, the AI was used to identify and integrate relevant scholarly references published between 2005 and the present to support the arguments presented in the manuscript. The author provided the following prompt to the AI:

"Please provide references between 2005 and the present to support the arguments made in the attached paper, retaining the current ones, in APA style throughout the paper and alphabetized at the end of the paper."

The prompt was reworded and reentered to include references between 2021 and 2026 as suggested by a reviewer.

The AI tool was used to:

1. Suggest relevant peer-reviewed literature and scholarly sources to support existing arguments
2. Format all references according to MDPI style guidelines
3. Integrate citations appropriately within the text
4. Alphabetize the reference list
5. Remove journal references prior to 2021 and to retain all book references regardless of publication date

Following the use of this tool, the author reviewed and edited the content as needed and takes full responsibility for the content of the published article. All AI-suggested references were verified by the author for relevance, accuracy, and appropriateness to the manuscript's arguments. The author confirms that the use of AI did not compromise the academic integrity or originality of the work.

AI Tool: Claude 4.5 Sonnet (Anthropic)

Date of Use: January 7, 2026; February 5, 2026

Purpose: Literature review support and reference formatting

Specific Tasks Performed:

- Identification of peer-reviewed scholarly literature published between 2005 and 2025 relevant to genomic medicine, regulatory frameworks, individual autonomy, genetic surveillance, informed consent, genetic discrimination, commodification of genetic information, and governance models
    - Amended to 2021 2026 date range

- Integration of approximately 60 additional citations into the manuscript text to support existing arguments and theoretical frameworks
- Formatting of all in-text citations and references according to MDPI style guidelines
- Alphabetization and organization of the complete reference list
- Verification of citation accuracy and consistency throughout the manuscript

Author's Role and Verification:

Following AI assistance, the author independently reviewed all suggested references for scholarly rigor, relevance to the manuscript's arguments, accuracy of citation details, and appropriateness of integration within the text. The author verified the existence and content of cited sources, ensured that citations accurately represented the referenced works, and made editorial decisions about which suggestions to retain, modify, or exclude. The author maintains full intellectual responsibility for all claims, interpretations, and conclusions presented in the manuscript. The core arguments, theoretical framework, analysis, and original contributions of this work were developed entirely by the author without AI assistance.
